# Reliability and interobserver variability of a grading system of ventricular distension in dogs

**DOI:** 10.3389/fvets.2023.1271545

**Published:** 2023-11-23

**Authors:** Adriana Czerwik, Martin Jürgen Schmidt, Agnieszka Olszewska, Steven Hinz, Kathrin Büttner, Daniela Farke

**Affiliations:** ^1^Department of Veterinary Clinical Sciences, Small Animal Clinic, Neurosurgery, Neuroradiology and Clinical Neurology, Justus Liebig University, Giessen, Germany; ^2^Small Animal Veterinary Practice Westpark, Wettenberg, Germany; ^3^Unit for Biomathematics and Data Processing, Faculty of Veterinary Medicine, Justus Liebig University, Giessen, Germany

**Keywords:** canine hydrocephalus, magnetic resonance imaging, VPS, grading system, ventriculomegaly

## Abstract

**Introduction:**

Internal hydrocephalus is the most common malformation of the central nervous system in dogs. Although the grades of ventricular distension have importance for long-term prognosis, there is no standard classification scheme describing the grade of the ventricular distension in dogs.

**Materials and methods:**

Magnetic resonance imaging (MRI) scans from 147 dogs of various breed, sex, skull conformation, and weight were reviewed retrospectively and blinded between three observers. Based on objectively assessable morphologic characteristics, the lateral cerebral ventricles were graded as normal, minimally, mildly, moderately, severely enlarged or end stage (grade 0 to grade 5), respectively. Evans’ index or the ventricle brain index was also measured in all animals. Interobserver agreement between a very experienced, experienced, and unexperienced person was evaluated by the Spearman coefficient and kappa tests. Additionally, correlation to the ventricle brain index was determined using the Spearman coefficient and F-tests.

**Results:**

The Spearman correlation coefficient reached a very strong correlation (*r* = 0.97) between the experienced and very experienced observer and a strong correlation (*r* = 0.91) between the very experienced and unexperienced observer. The kappa value revealed excellent interobserver agreement between the very experienced and experienced observers (weighted kappa 0.91) and moderate between the very experienced and unexperienced observers (weighted kappa 0.75). The ventricular-brain index correlated (*r* = 0.94, Spearman coefficient test) with the grading system, indicating that a more elevated ratio was related to a more advanced degree of ventricular enlargement. The interobserver agreement with regard to the grade between the neurologist in training and a board-certified neurologist was excellent and between the board-certified neurologist and general practitioner achieved lower values.

**Conclusion:**

The presented MRI-based grading of ventricular enlargement is a reliable and functional method for an objective grading of the ventricular system in dogs. Some experience in MRI and brain anatomy is needed for interpretation and grading.

## 1 Introduction

Internal hydrocephalus is the most common malformation of the central nervous system in dogs, which can be successfully treated using ventriculoperitoneal shunting (VPS) (1). Since the introduction of the technique to veterinary neurosurgery in the 1960s (2), a number of studies have been conducted that described structural characteristics of the hydrocephalic brain indicating surgical therapy (3), documented changes in intraventricular pressures (4), VPS associated complications (5, 6), long-term survival (7), and success in terms of improvement of clinical signs (8). Although this information helps to optimize neurosurgical treatment, variation in surgical care and decision-making remains common in veterinary medicine. Refining patient selection and tailoring appropriate treatment to each individual animal is a further important step in improving treatment. In that respect, grading of ventricular distension has important implications for the appropriate surgical technique (9), the occurrence of overshunting related complications (6) and, potentially, on the prediction of clinical effectiveness of shunting. In order to compare results of surgical interventions performed in multiple institutions, a clear classification of structural changes of hydrocephalic brains would be necessary. Currently, ventricular distention is mostly subjectively graded without any standards that define the different grades (1, 10, 11). An objective and easy grading system is necessary that classifies ventricular distension with high reproducibility. It should not require high-level expertise or the need of time-consuming image postprocessing. In this study, we propose a grading system for the classification of ventricular distension in a large cohort of dogs and assess its interobserver variability.

## 2 Materials and methods

### 2.1 Animals

The database of MRI scans of the Department of Veterinary Clinical Sciences, Small Animal Clinic, Neurosurgery and Clinical Neurology at the Justus Liebig University (Giessen, Germany), was retrospectively searched for MRI reports of the brain including the diagnoses “internal hydrocephalus,” “ventriculomegaly,” “dilated ventricles,” or “without special findings.” The sex, age, and body weight of the dogs at the time of scanning were recorded. Approval from the ethics committee of the Justus Liebig University was not sought as retrospective studies of images and records stored in the archive are not subject to ethical review.

### 2.2 Magnetic resonance imaging

Imaging was performed using a 1.5 (Phillips Intera Gyroscan, Philips Healthcare, Hamburg Germany) or a 3.0 Tesla high field MRI scanner (Siemens Magnetom Verio, Erlangen Germany). Images included at least sagittal, transverse, and dorsal T2-weighted images (Turbo Spin Echo, TR 2,900 ms, TE 120 ms, slice thickness 3 mm). The field of view measured 180 × 180 mm in small dogs and 210 × 210 mm in large dogs. The matrix was 288 × 288 in small dogs and 384 × 384 in large dogs, leading to an in-plane pixel size between 0.625 × 0.625 mm and 0.54 × 0.54 mm.

### 2.3 Image analysis

**Figure 1:**
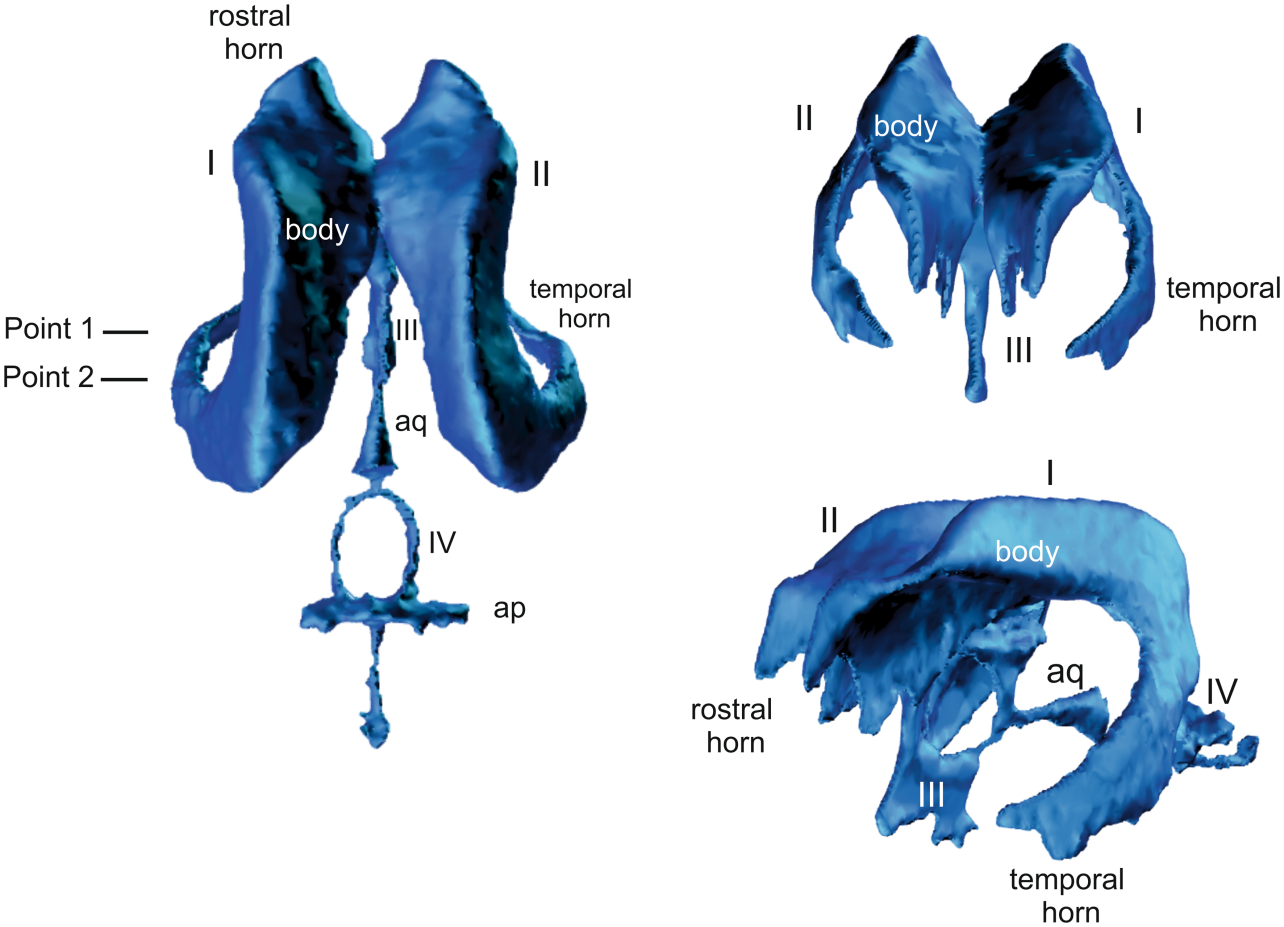
3D model of the canine ventricular system showing the plane of the. I and II = lateral ventricles, III = third ventricle, IV = fourth ventricle, aq = cerebral aqueduct.

A neurologist in training (AC), a board-certified neurologist with long experience in treatment of internal hydrocephalus (DF), and a general practitioner without MRI experience (SH) analyzed the MR-images. The general practitioner did not receive any previous training in MRI interpretation or brain anatomy. All interpreters were asked to define the grade of ventricular distension based on morphological characteristics assessed in transversal images at two defined measuring points:

**Point 1 (P1):** at the caudal aspect of the third ventricle where the commissure of the fornix connects with the corpus callosum (Figure 1).

**Point 2 (P2):** at the level of the rostral colliculi (Figure 1).

**Figure 2:**
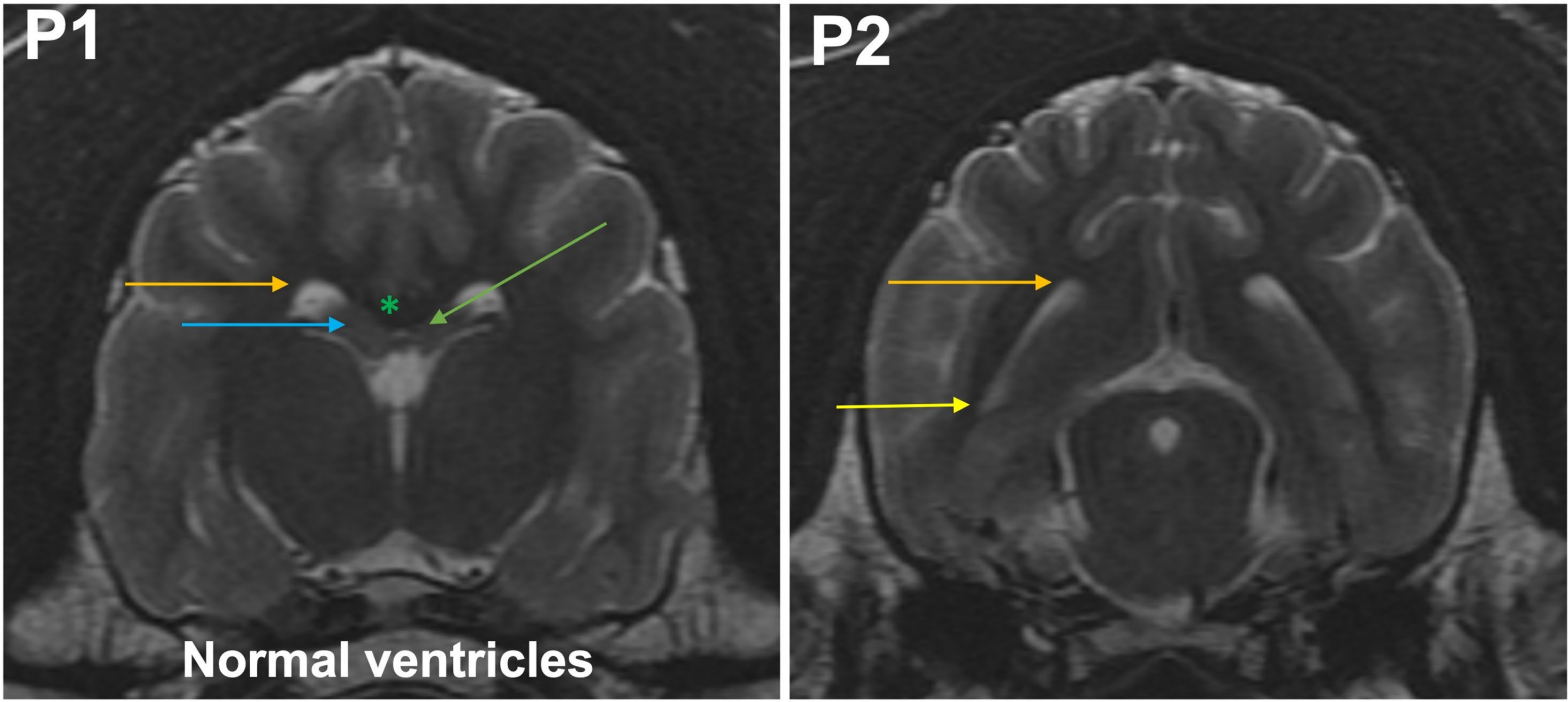
T2-weighted MR images of the brain in the transverse plane (P1, P2) showing normal ventricles. P1, the bodies of the lateral ventricles have a drop-like or triangular shape (orange arrow). The corpus callosum (green asterisk), hippocampus (blue arrow), and fornix (green arrow) rest on the dorsal thalamus. Temporal horn is not seen as a CSF filled cavity. At P2, the body (orange arrow) and temporal horn (yellow arrow) have equal dimensions.

Characteristics of the ventricular system were defined as follows:

**Grade 0 (normal lateral ventricles):** at P1, the bodies of the lateral ventricles have a drop-like or triangular shape. The corpus callosum, hippocampus, and fornix rest on the dorsal thalamus. Temporal horn is not seen as a CSF filled cavity (Figures 1, 2). At P2, the body and temporal horn have equal dimensions.

**Figure 3:**
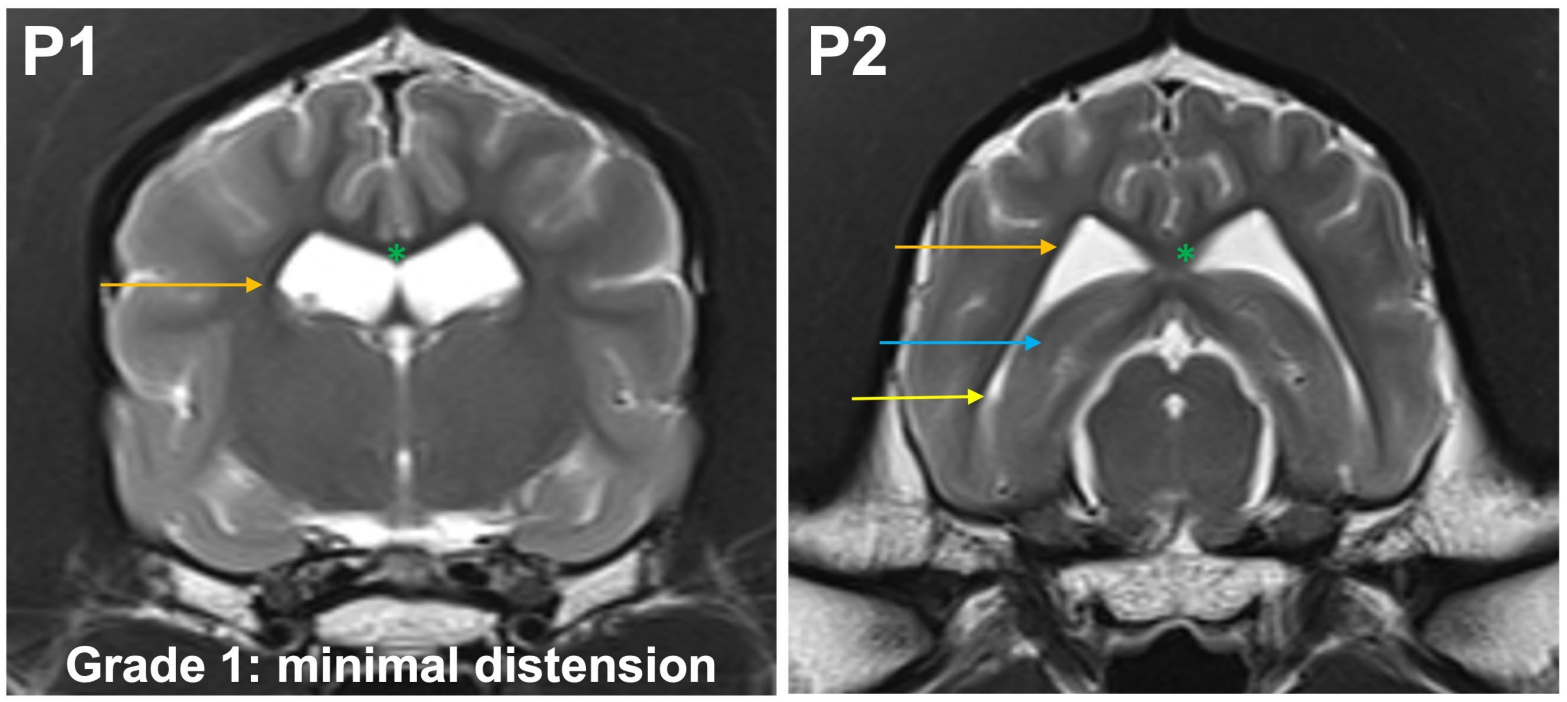
T2-weighted MR images of the brain in the transverse plane (P1, P2) showing grade 1 distension. The body of the lateral ventricle (orange arrow) has changed from drop-shaped to a round/oval cross section. The temporal horns are not visible at P1. The commissure of the fornix and corpus callosum are joined (green asterisk). At P2, the temporal horn (yellow arrow) is dilated and a CSF signal is seen. Maximal mediolateral extension of the temporal horn (cave: not of the body of the lateral ventricle) does not exceed the dimensions of the hippocampus (blue arrow) in that plane.

**Grade 1 (minimally):** the body of the lateral ventricle has changed from drop-shaped to a round/oval cross section. The temporal horns are not visible at P1. The commissure of the fornix and corpus callosum are joined. At P2, the temporal horn is dilated and a CSF signal is seen. Maximal mediolateral extension of the temporal horn (cave: not of the body) does not exceed the dimensions of the hippocampus in that plane (Figure 3).

**Figure 4:**
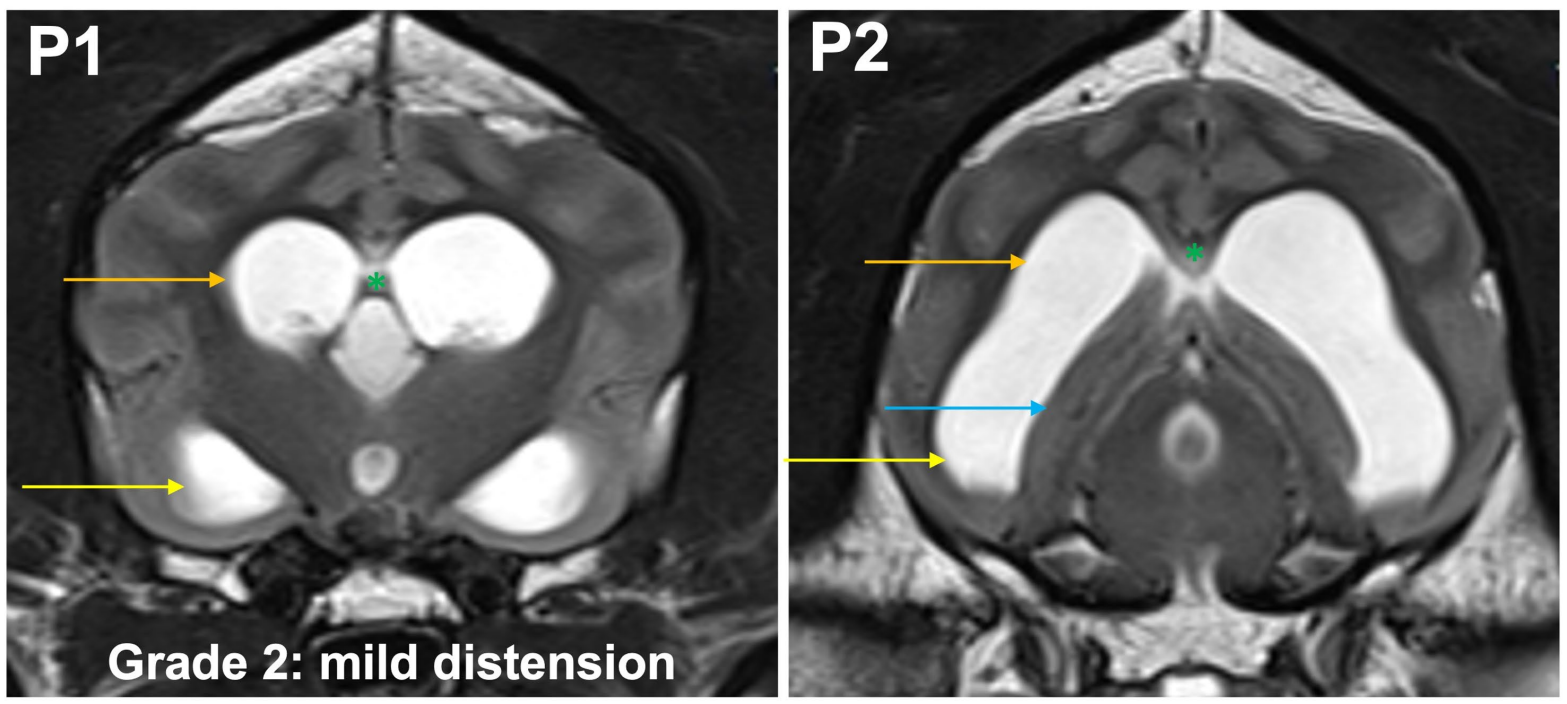
T2-weighted MR images of the brain in the transverse plane (P1, P2) showing grade 2 distension. The temporal horns (yellow arrow) are dilated and visible at P1 and P2. The maximal mediolateral extension of the temporal horn exceeds the dimensions of the hippocampus (blue arrow) at P2, the maximal mediolateral extension of the body (orange arrow) is the same or larger than the temporal horn at P2. The commissure of the fornix and the corpus callosum are joined (green asterisk).

**Grade 2 (mildly):** the temporal horns are dilated and visible at P2 and P1. The maximal mediolateral extension of the temporal horn exceeds the dimensions of the hippocampus at P2, the maximal mediolateral extension of the body is the same or larger than the temporal horn at P2. The commissure of the fornix and the corpus callosum are joined (Figure 4).

**Figure 5:**
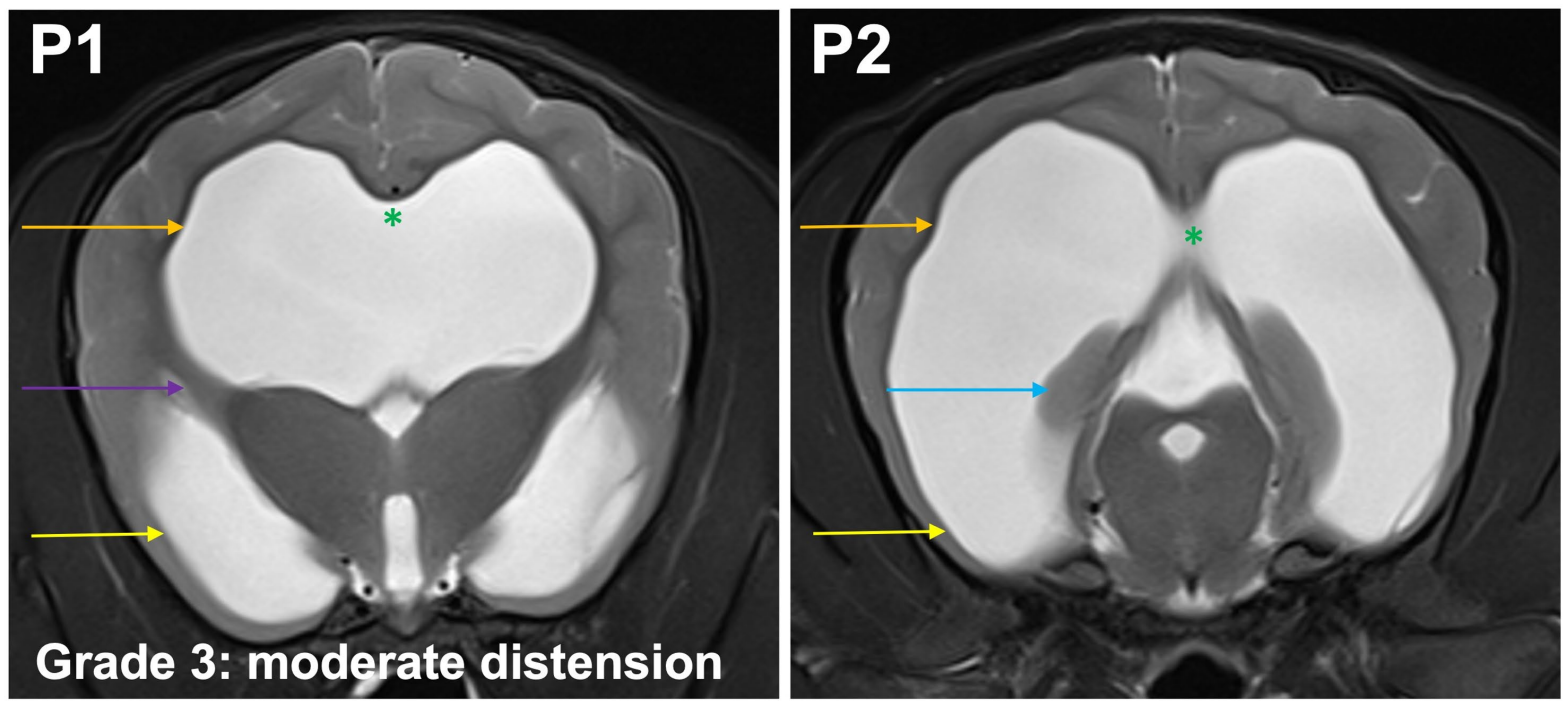
T2-weighted MR images of the brain in the transverse plane (P1, P2) showing grade 3 distension. The temporal horns (yellow arrow) are dilated at P1 and P2, the maximal mediolateral extension of the lateral ventricles (orange arrow) exceeds the dimensions of the hippocampus (blue arrow) at P2. Convergence of the fornix and corpus callosum is lost (green asterisk). The optic radiation connects the subcortical white matter to the diencephalon and separates the temporal horn from the body at P1 (purple arrow). Sulci and gyri with visible white matter extensions into the gyrus are present.

**Grade 3 (moderately):** the temporal horns are dilated at P2 and P1, the maximal mediolateral extension of the lateral ventricles exceeds the dimensions of the hippocampus at P2. Convergence of the fornix and corpus callosum is lost. The optic radiation connects the subcortical white matter to the diencephalon and separates the temporal horn from the body at P1. Sulci and gyri with visible white matter extensions into the gyrus are present (Figure 5).

**Figure 6:**
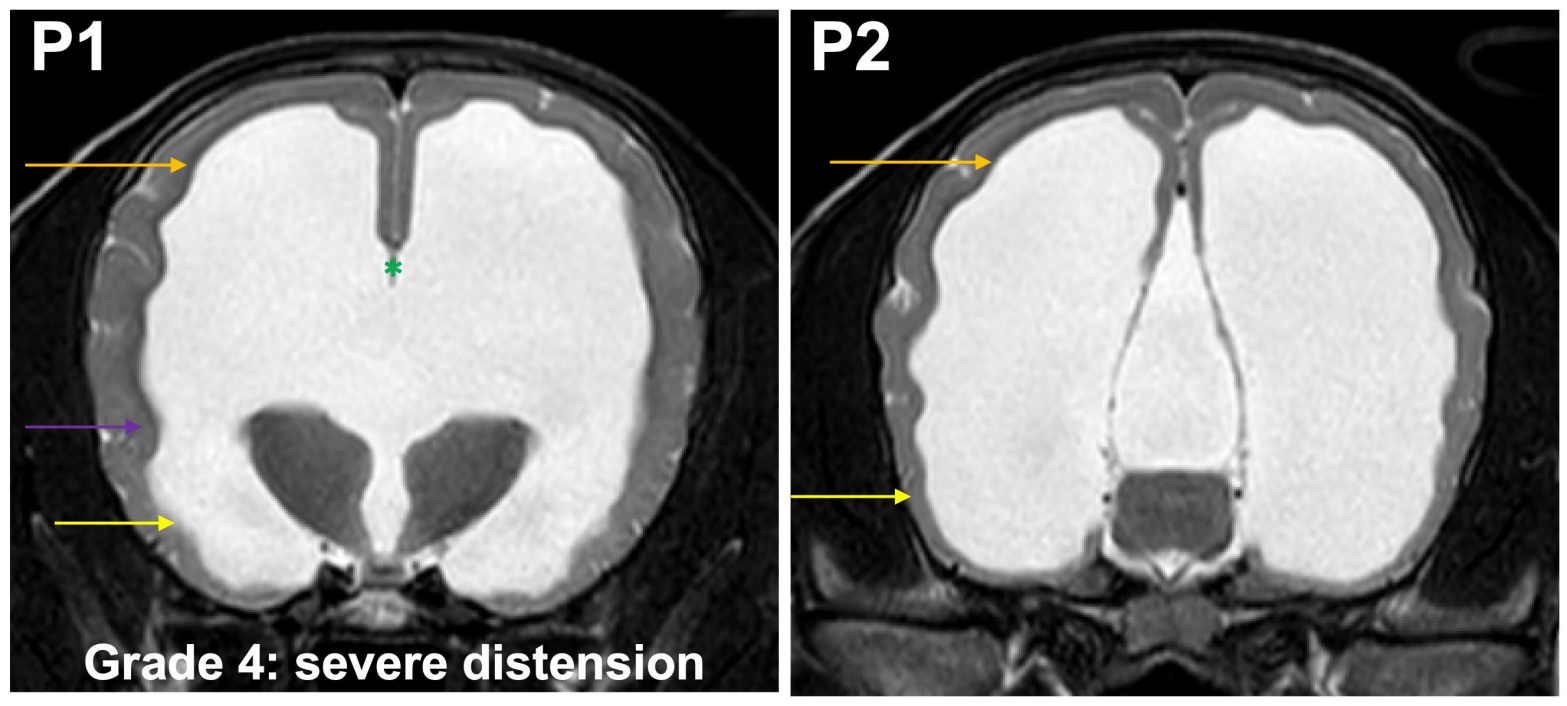
T2-weighted MR images of the brain in the transverse plane (P1, P2) showing grade 4 distension. Temporal horns (yellow arrow) are dilated at P1 and P2, the cross section of the ventricles exceeds the dimensions of the hippocampus at P2, which is no longer visible, the body (orange arrow) and temporal horn have more or less equal dimensions. Convergence of the fornix and corpus callosum is lost (green asterisk). The optic radiation does not connect the subcortical white matter to the diencephalon (purple arrow). The gyri are flat, sulci are shallow.

**Grade 4 (severely):** temporal horns are dilated at P2 and P1, the cross section of the ventricles should exceed the dimensions of the hippocampus at P2 which is no longer visible at this point, the body and temporal horn have more or less equal dimensions. Convergence of the fornix and corpus callosum is lost. The optic radiation does not connect the subcortical white matter to the diencephalon (Figure 6). The gyri are flat, sulci are shallow.

**Figure 7:**
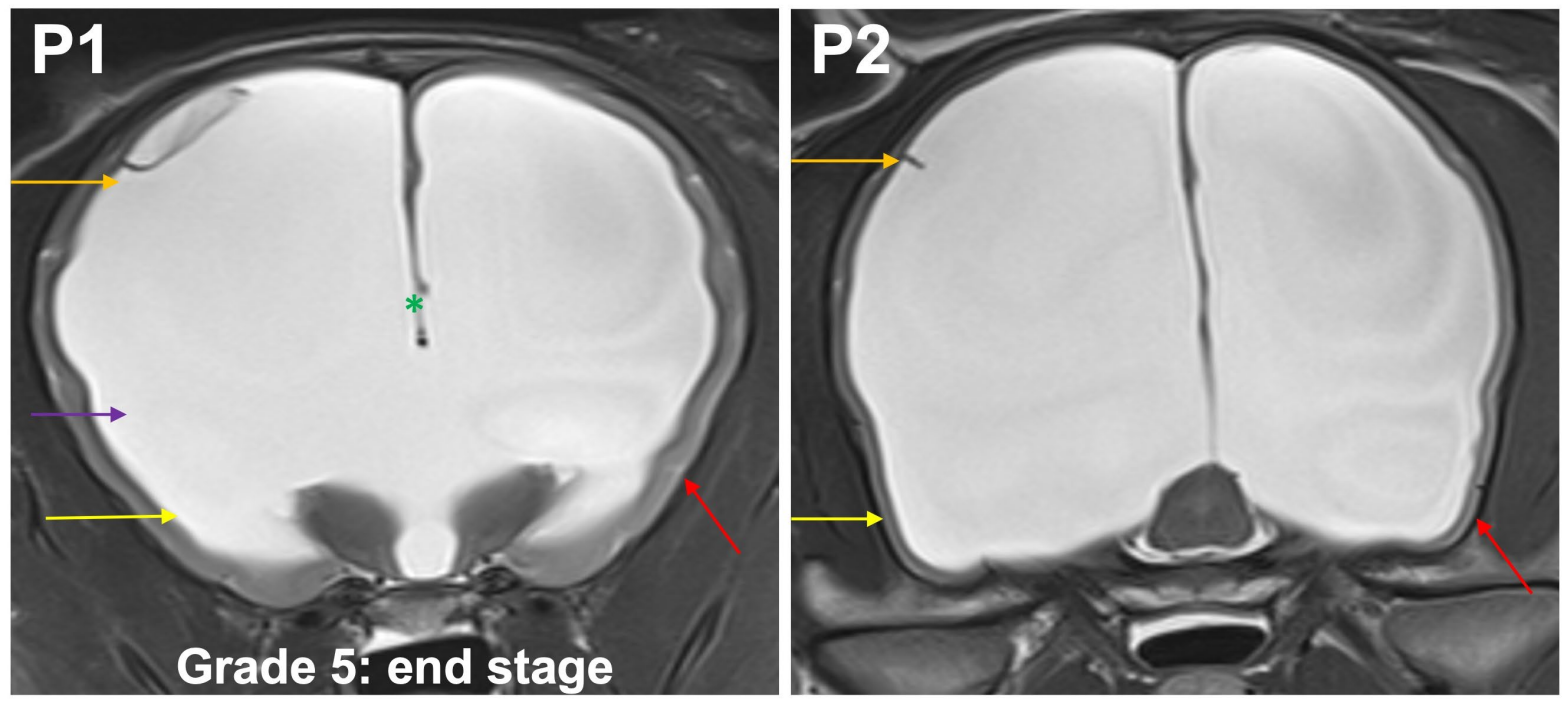
T2-weighted MR images of the brain in the transverse plane (P1, P2) showing grade 5 distension. Optic radiation does not connect subcortical white matter to the diencephalon (purple arrow); the temporal horns (yellow arrow) and body (orange arrow) form a continuous space. Convergence of the fornix and corpus callosum is lost (green asterisk). Only very short sulci, but no gyri (red arrow) with white matter extensions from the subcortical white matter are present in the entire neocortex.

**Grade 5 (end stage):** optic radiation does not connect subcortical white matter to the diencephalon; the temporal horns and body form a continuous space. Convergence of the fornix and corpus callosum is lost. Only very short sulci, but no gyri with white matter extensions from the subcortical white matter are present in the entire neocortex (Figure 7).

### 2.4 Ventricle brain index

The ventricle/brain index was determined in all image sets by measuring the maximum continuous distance between the internal borders of the ventricles divided by the maximum width of the brain parenchyma in the same image in that dorsal plane, in which the lateral cerebral ventricles have the largest dimensions (3).

### 2.5 Statistical analysis

Statistical analysis was performed using a commercial statistical software package (Base SAS^®^ 9.4 Procedures Guide: Statistical Procedures, 2nd edition ed. Statistical Analysis System Institute Inc., Cary, NC, United States). The level of ventricle enlargement based on the grading system was assessed and a confidence level of 95% determined. Agreement between observers was evaluated using the Spearman coefficient and kappa tests. Comparison to VBI was performed by a board-certificated neurologist. The data for grading and VBI measurements were nearly normally distributed. Spearman’s rank correlation was used to analyze the relation between the grading of the most experienced observer (DF) and VBI as a metrical value. Furthermore, a univariant analysis of variance was performed using the grading of the most experienced observer as a fixed variable and the VBI measurement as a dependent variable. Furthermore, an F-test was performed on the results to test for significance.

## 3 Results

### 3.1 Animals

One hundred forty-seven dogs were included in the study, with a median age of 24 months (range: 1–168 months) and median body weight of 8.6 kg (range: 0.7–58 kg). The breeds included in the study are summarized in Table 1.

**Table 1:** Breeds of dogs included in the study.

Breed	Number (147)
Chihuahua	23
Mix-breed dogs	21
Cavalier King Charles Spaniel	13
Australian Shepherd, Labrador Retriever, French Bulldog, English Bulldog	7 each
Rhodesian Ridgeback, Border Collie, Golden Retriever, German Shepherd	4 each
Jack Russell terrier, Mini Bull terrier, Papillon	3 each
Pug, Belgian Malinois, Austrian Black and Tan Hound, Boxer, Peruvian Hairless Dog, Russian Toy, Maltese, American Staffordshire terrier, American Cocker Spaniel, Dachshund, Beagle	2 each
Tibetan Spaniel, Rottweiler, Miniature Schnauzer, West Highland white terrier, Boston terrier, Yorkshire terrier, Keeshond, Havanese, German Pincher, Brussels Griffon, Entlebucher Mountain Dog, Flat-Coated Retriever, German Wirehaired Pointer, Siberian Husky	1 each

### 3.2 Interobserver variability

**Figure 8:**
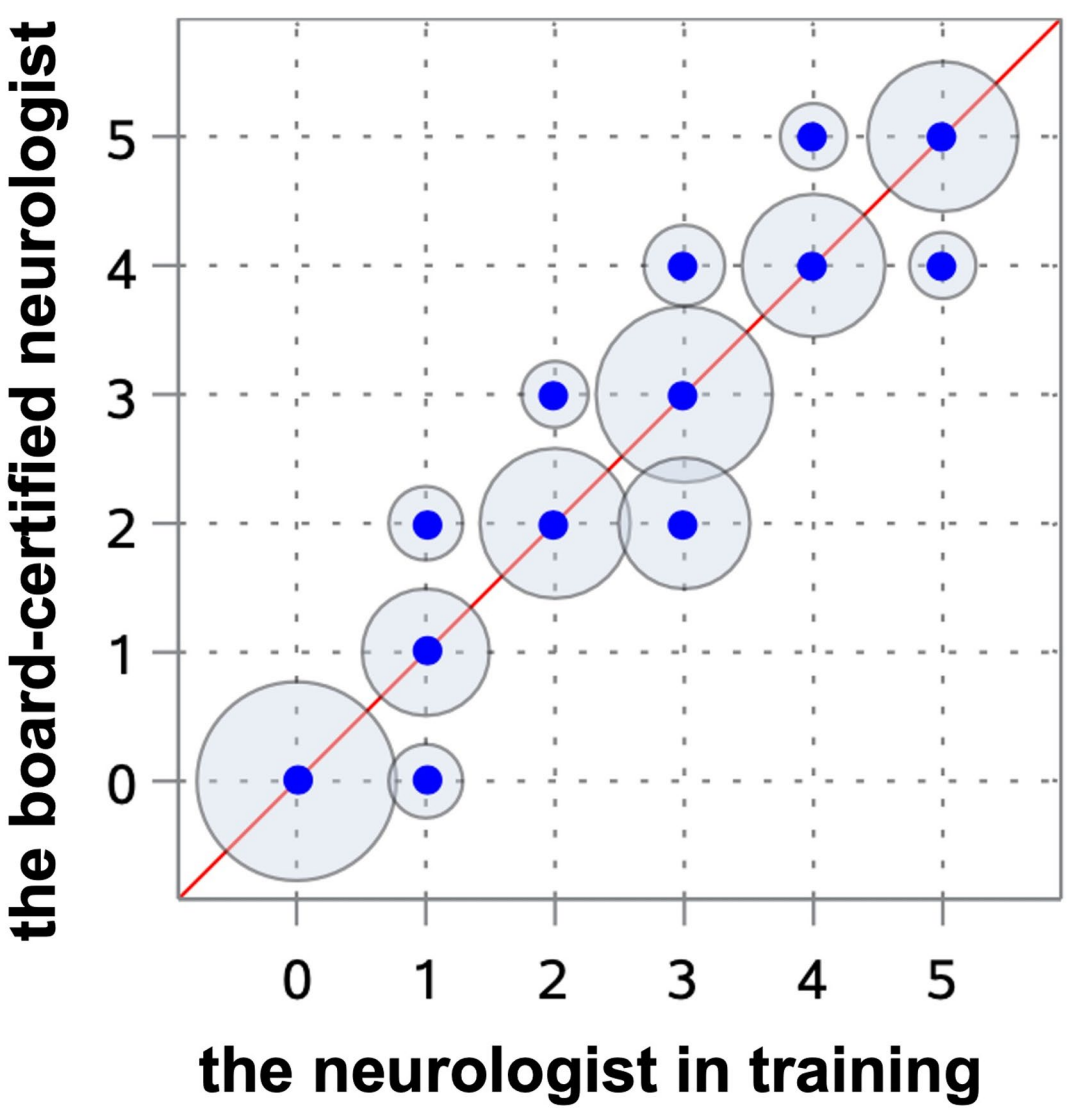
Scatterplot representing the Spearman’s correlation coefficient between the neurologist in training and the board-certified neurologist. The blue points show the agreement of the different gradings between both observers. The circles around the dots indicate the frequency of the observed agreement, the more observations are made with an agreement the bigger the circle around the blue point.

**Figure 9:**
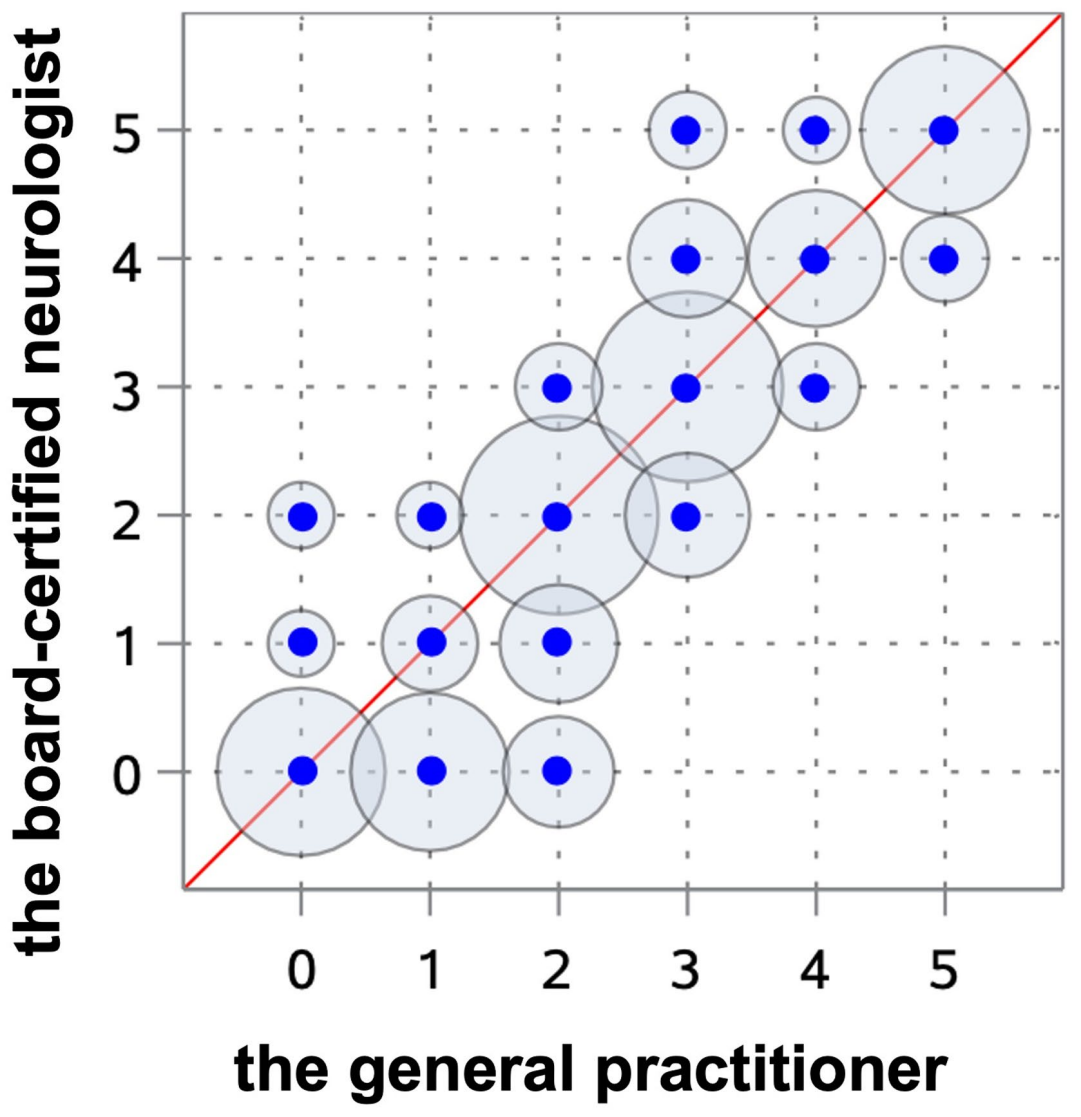
Scatterplot representing the Spearman’s correlation coefficient between the board-certified neurologist and general practitioner. The blue points show the agreement of the different gradings between both observers. The circles around the dots indicate the frequency of the observed agreement, the more observations are made with an agreement the bigger the circle around the blue point.

All observers were able to grade the ventricular dimensions based on the described characteristics. A comparison of interobserver assessment is summarized in Table 2. The Spearman correlation coefficient reached a very strong correlation (*r* = 0.97) (Cl 95% 0.95–0.98) (*p* < 0.0001) between the neurologist in training and the board-certified neurologist (Figure 8), and even a strong correlation (*r* = 0.91) (Cl 95% for 0.88–0.94) (*p* < 0.0001) between the board-certified neurologist and general practitioner (Figure 9). Cohen’s kappa coefficient revealed excellent interobserver agreement between the neurologist in training and the board-certified neurologist (weighted kappa = 0.91) (Cl 95% 0.88–0.95) and moderate between the board-certified neurologist and general practitioner (weighted kappa = 0.75) (Cl 95% 0.69–0.81). The overall interobserver agreement with regard to grade 0–5 between the neurologist in training and the board-certified neurologist was excellent, except for grade 2 and 3 (Table 2). The interobserver agreement between the board-certified neurologist and general practitioner achieved lower values (Table 2). Spearman’s correlation showed a high correlation (*r* = 0.94) (Cl 95% 0.92–0.96) (*p* < 0.0001) between the increasing grades and increasing VBI for the most experienced observer. Analysis of variance also demonstrated *r*^2^ of 0.9, indicating that 90% of the variance of the studied VBI can be explained by the variance of the adjusted grading by an experienced observer (*F* = 305.67; *p* < 0.0001). There was a considerable overlap between VBIs between the different grades (Table 2).

**Table 2:** Classification of the ventricular distension as grade 0 to grade 5 in all 147 dogs by each assessor as well as minimum to maximum values and mean values of VBI evaluated by board-certified neurologist.

Grade	a board-certified neurologist	a neurologist in training	a general practitioner	VBI minimum–maximum	VBI mean (standard deviation)
0	36	34	18	0.15–0.41	0.30 (0.08)
1	12	16	19	0.33–0.55	0.43 (0.07)
2	33	19	39	0.38–0.76	0.61 (0.09)
3	27	42	38	0.56–0.79	0.72 (0.06)
4	20	17	14	0.64–0.93	0.84 (0.07)
5	19	19	19	0.83–0.94	0.91 (0.03)

## 4 Discussion

This is the first investigation in veterinary medicine regarding a classification scheme describing 5 grades of ventricular distension in dogs. The investigated system showed a low interrater variability between the neurologist in training and the board-certified neurologist. However, grades 2 and 3 were difficult to differentiate in some images, resulting in a low reliability of these grades (Table 2). The reason for this inconsistency remains unclear. In order to keep the grading simple, the dimensions of the hippocampus and temporal horn were visually assessed. It is possible that variability would decrease if measurement tools were used to quantify these brain structures. The agreement between the board-certified neurologist and the general practitioner was only moderate among all grades, except grade 5 (Table 2). The general practitioner explained that his limited knowledge of brain morphology made it difficult for him to find the proper image plains and even localize structures like the hippocampus or corpus callosum. Therefore, a proper grading of the ventricular system seems to need at least some experience of the observer regarding brain morphology and MRI interpretation.

Surgical treatment for internal hydrocephalus continues to develop. The number of involved ventricles, as well as the amount of ventricular distension is relevant for the outcome of VPS as these findings are associated with a higher risk of hemispheric collapse and subdural hemorrhage (6). One important goal for the future treatment of hydrocephalus would be the development of a success score that allows prediction of which animal will benefit from VPS or not and which animal is likely to develop complications. The presented grading system has the potential of being useful for the definition of one important factor in such scoring: the ventricular dimensions.

Our experience with grading of ventricular dimensions of animals referred to our clinic or from radiologic assessment in our own hospital has shown that the assessment of ventricle dimensions is highly subjective in general. There is a special bias in the assessment of ventricular dimensions of brachycephalic dogs compared to mesocephalic dogs. Enlarged ventricular dimensions often get lower subjective grades in brachycephalic dogs, probably because small brachycephalic dogs frequently show some degree of ventricular enlargement, referred to as “ventriculomegaly.” Ventriculomegaly was for a long time assessed as clinically irrelevant (3, 12, 13). However, it was shown that white matter atrophy and a reduced cerebral blood flow occurs in brachycephalic dogs having “breed specific” enlarged lateral ventricles, which implies abnormal CSF drainage from the ventricular system and negative effects of ventricular distension on cerebral white matter (14, 15). These findings imply that the term “ventriculomegaly” should be avoided and the situation be assessed as ventricular enlargement of lower grades. It is currently under evaluation if white matter atrophy and reduced white matter perfusion are associated with cognitive problems in dogs as in humans (16–18). This grading scheme helps to make the assessment of ventricular enlargement more objective among different breeds and to categorize ventricular size by using standardized anatomical structures. Although brain structures differ between brachycephalic and mesocephalic breeds, the planes chosen for evaluation (P1 and P2) are less affected by the subsequent shortening and rotation of brachycephalic head shapes than very rostral (e.g., olfactory bulb) or very caudal anatomical structures (e.g., craniocervical junction). However ventricular enlargement is a common finding of brachycephalic dogs and result of the skull shape variation (19).

Evans’ index or the VBI was previously used to grade ventricular enlargement and as an indirect evaluation method for ventricular system distention in neurosurgery in human medicine (20). However, the Evans’ index describes a linear model of continuous ventricular enlargement, but it does not resemble a classification system as measurements are performed routinely at one anatomical point. A classification, as used in human medicine, concentrates on more parts of the ventricular system and is able to evaluate the whole ventricular space and its distention (20, 21). As a matter of fact, the interobserver variability of the Evans’ index was never confirmed (21, 22). In this study, we observed a considerable overlap of VBI in relation to the different grades. The VBI was calculated and correlated with the grading system by the certified neurologist. The mean VBI for grade 0 was 0.30 and 0.91 for grade 5 (Table 2). Although our results showed that the higher VBI, the greater the ventricular dilatation, the same VBI was often measured in three different grades, which makes it a rather unreliable parameter for an unequivocal grading system. The reason for this range is unclear. Interobserver differences for calculating Evans’ index were also found in studies on hydrocephalic humans (19). It was found to vary greatly depending on the location and angle of the slice of the CT or MRI at which the frontal horns and maximal inner skull diameters are measured (21, 22). In order to determine VBI, it is necessary to set CSF and brain parenchyma boundaries, which are not always clear cut (21, 22). Given this imprecision, VBI seems rather inappropriate to precisely grade ventricular distension. But as there is a considerable overlap between our graduation and VBI, further studies are needed to test both variables for their correlation with clinical and outcome measures.

One limitation of this study is the presence of only one observer in each of the 3 different experience groups. This is enough to assess the feasibility of the above described grading system, but a correlation between more observers with the same experience level would be helpful to evaluate the reliability of this new grading system among different experience levels. Another possibility would be an interobserver assessment of the neurologist in training and the general practitioner after some training in MRI interpretation and brain anatomy. In consequence interobserver agreement could improve, if this is not the case definitions of specific grades might be specified to increase the agreement. Further validation by intraobserver evaluation to provide evidence of consistency, more interobserver measurements at different experience levels and whether training will improve reliability should be performed on a larger cohort should be performed. Another limitation is that the correlation between the degree of the ventricular enlargement and the clinical symptoms or other MRI findings like periventricular edema or deformation of the interthalamic adhesion has not been evaluated, therefore we cannot conclude whether the grading system has good clinical dependence and relevance. The decision about surgical VPS implantation should be considered based on the individual clinical symptoms, progression of clinical signs and concurrent diseases. The risk of ventricular collapse is very high in case of very advanced dilatation of the ventricles, which reflects a grade 5 in this study (6). In case with grade 1–2 ventricular enlargement, the ventricular enlargement might be without clinical importance (8), but further studies are warranted. However further validation of VBI and this grading system are needed to guarantee a reliable assessment of ventricular enlargement and correlation to clinic and outcome measurements.

## 5 Conclusion

The findings of the study allow us to conclude that the system described in this study is a functional method for an objective grading of the ventricular system in dogs but requires basic neuroanatomical knowledge. Although the present grading system might have some inaccuracy in the lower grades, it represents an improvement compared to former methods. Further evaluation of this grading scheme with regard to inter- and intra-observer agreement and to clinical and outcome measurements are needed.

## Data availability statement

The original contributions presented in the study are included in the article/supplementary material, further inquiries can be directed to the corresponding author.

## Ethics statement

Ethical approval was not required for the study involving animals in accordance with the local legislation and institutional requirements because only retrospective data from MRI archives were examined, which are not subject to ethical approval according to the German Animal Protection Act.

## Author contributions

AC: Data curation, Formal analysis, Methodology, Writing – original draft, Writing – review & editing. MS: Supervision, Writing – review & editing. AO: Writing – review & editing. SH: Data curation, Methodology, Writing – review & editing. KB: Data curation, Methodology, Software, Writing – review & editing. DF: Data curation, Methodology, Supervision, Writing – review & editing.
